# How Machine Perfusion Ameliorates Hepatic Ischaemia Reperfusion Injury

**DOI:** 10.3390/ijms22147523

**Published:** 2021-07-14

**Authors:** George Clarke, Hynek Mergental, Angus Hann, M. Thamara P. R. Perera, Simon C. Afford, Darius F. Mirza

**Affiliations:** 1 Liver Unit, Queen Elizabeth Hospital Birmingham, Birmingham B15 2TH, UK; George.Clarke@uhb.nhs.uk (G.C.); Hynek.Mergental@uhb.nhs.uk (H.M.); Angus.Hann@uhb.nhs.uk (A.H.); Thamara.Perera@uhb.nhs.uk (M.T.P.R.P.); 2 Birmingham Biomedical Research Centre, National Institute for Health Research (NIHR), University of Birmingham and University Hospitals Birmingham NHS Foundation Trust, Birmingham B15 2TH, UK; s.c.afford@bham.ac.uk; 3 Centre for Liver and Gastrointestinal Research, Institute of Immunology and Immunotherapy, University of Birmingham, Birmingham B15 2TH, UK

**Keywords:** machine perfusion, normothermic, hypothermic, ischaemia reperfusion injury, liver transplant

## Abstract

The increasing disparity between the number of patients listed for transplantation and the number of suitable organs has led to the increasing use of extended criteria donors (ECDs). ECDs are at increased risk of developing ischaemia reperfusion injury and greater risk of post-transplant complications. Ischaemia reperfusion injury is a major complication of organ transplantation defined as the inflammatory changes seen following the disruption and restoration of blood flow to an organ—it is a multifactorial process with the potential to cause both local and systemic organ failure. The utilisation of machine perfusion under normothermic (37 degrees Celsius) and hypothermic (4–10 degrees Celsius) has proven to be a significant advancement in organ preservation and restoration. One of the key benefits is its ability to optimise suboptimal organs for successful transplantation. This review is focused on examining ischaemia reperfusion injury and how machine perfusion ameliorates the graft’s response to this.

## 1. Introduction

Liver transplantation remains the only definitive curative intervention for individuals with end-stage liver disease [1]. There is a growing disparity between the increasing number of patients listed for transplant and the limited number of organs suitable for transplantation, which is reflected in the morbidity and mortality of patients awaiting this life-extending procedure. The length of time between being added to the transplant waiting list and undergoing a liver transplant (median time of 99 days between 2015–2018) means that a significant proportion of patients (15–20%) die before they receive a graft [2,3].

The pressure to source suitable organs has led to the increasing use of extended criteria donors (ECDs). ECD grafts are associated with an increased risk of primary non-function or initial poor function. Factors that determine if a graft is suboptimal, and thus, ECD are; donor aged 60 years or above, steatosis >30%, positive hepatitis B and C virologies, cold ischaemic time in excess of 12 h, donation after circulatory death (DCD), split-liver grafts, and living-related donations [4]. 

Ischaemia is defined as the disruption of cellular metabolism due to poor oxygenation secondary to the inadequate supply of blood to bodily tissue. The restoration of blood flow to previously ischaemic tissue is termed reperfusion. This is a corrective process that is complicated by the accumulation of anaerobic by-products and tissue injury, which is termed ischaemia reperfusion (I/R) injury. I/R injury may occur as a result of numerous interventions including hepatic resection, transplantation, and traumatic injury. It is a major complication of liver transplantation, and further research is required to reduce its impact on surgical outcomes.

The gold standard of organ preservation at present remains static cold storage (SCS), a process which relies on the storage and transportation of the organ on ice, infused with specialist fluid, to reduce cellular metabolism and injury. This technique has been used for decades and works well in good quality donors. Despite these numerous methods, cold ischaemic injury remains a reality—the results of which are even more pronounced in marginal livers [5]. 

Machine perfusion is a novel method of organ preservation that facilitates the preservation and optimisation of organs under variable temperatures. This technique has seen great success in kidney transplantation, where it was initially developed, and over the past decade has become increasingly prevalent in the optimisation of liver grafts prior to transplantation with significant clinical success [6,7]. 

This review will discuss the pathophysiology of ischemic reperfusion injury and how machine perfusion ameliorates this injury.

## 2. Ischaemia Reperfusion Injury

I/R injury is a multifocal process involving numerous cell types and signalling pathways. It is an enormously complex process and, as such, its pathophysiology is not entirely understood. Great strides have been taken over the preceding decades to further evaluate the process and theorise modalities to augment it. I/R injury in its most simple form consists of an initial ischaemic phase followed by a reperfusion phase. Reperfusion can be further divided into an early and late phase.

During the initial ischaemic period, there is reduced oxygenation at the cellular level leading to anaerobic metabolism within the various cell types that make up the liver architecture [8]. Anaerobic metabolism leads to intracellular acidosis following the accumulation of lactic acid and hydrogen ions (H^+^). Upregulation of the Na^+^/H^+^ transporter leads to the exchange of intracellular hydrogen ions for extracellular sodium ions. This shift in sodium ions leads to increased intracellular sodium concentrations, which ultimately results in cellular swelling, rupture, and death [9]. The dysfunction of the ATP-dependent Na^+^/H^+^ exchanger under anaerobic conditions further propagates increasing intracellular sodium concentrations.

An increase in intracellular calcium has been implicated in the early phase of I/R injury. The increase in intracellular sodium stimulates the Na^2+^/Ca^2+^ exchanger causing a rise in intracellular calcium. The loss of ATP inhibits Ca^2+^-ATPase in the membrane, further compounding the increase in intracellular calcium. Calcium propagates the injury seen in I/R disrupting mitochondria and stimulating calcium-dependent enzymes leading to apoptosis and necrosis [8,10,11,12]

### 2.1. Reactive Oxygen Species

Following the restoration of vascular inflow and reperfusion of the ischaemic tissue there is an acceleration in tissue damage that exceeds that seen in ischaemia alone, in which the production of reactive oxygen species (ROS) plays a large role. Hypoxanthine is a by-product of ATP degradation. Under normal conditions, hypoxanthine is oxidised to xanthine by an enzyme, xanthine dehydrogenase, however xanthine dehydrogenase is converted to xanthine oxidase under ischaemic conditions. Xanthine oxidase, as opposed to xanthine dehydrogenase, generates ROS. Xanthine oxidase requires oxygen as a substrate and during ischaemia is unable to convert hypoxanthine to xanthine and accumulates toxic levels of hypoxanthine in the tissue. Following the influx of oxygen during reperfusion, hypoxanthine is degraded to uric acid by xanthine oxidase, which is catalysed by oxygen. Superoxide anion (O_2_^−^) is produced and is converted to hydrogen peroxide (H_2_O_2_) and hydroxyl radical (OH·) [13]. 

O_2_^−^, H_2_O_2_, and OH· are all potent ROS which damage the lipid structures of cell membranes by lipid peroxidation via their oxidising and reducing properties [14]. Damage to the cell membranes leads to the production and release of proinflammatory eicosanoids, in addition to the loss of membrane integrity and cell death. ROS encourage the formation of arachidonic acid, an important substrate in the synthesis of eicosanoids (e.g., prostaglandins, thromboxane, and leukotrienes) [15]. The activation of endothelial cells upregulates the activity of the NF-κB stimulating leukocyte adhesion molecules.

### 2.2. Hepatic Microcirculation

Under normal conditions, the hepatic microcirculation is maintained by a balance between nitric oxide (NO) and endothelin (ET) [16]. The loss of this equilibrium induces vasoconstriction and narrowing of the sinusoidal lumen.

NO is synthesised from L-arginine by nitric oxide synthase (NOS), of which three isoforms have been identified [17]. The three isoforms of NOS are inducible (iNOS), endothelial (eNOS), and neuronal NOS (which has no identified role in I/R injury). The pathophysiology of NO is tissue-dependent and its role in I/R injury variable.

The initial surge of nitric oxide seen during the initial phase of ischaemia is credited to the activation of eNOS. This is followed by a decline in NO levels during reperfusion due to the decline in endothelial function and loss of functional eNOS. eNOS plays an important role in the modulation of vascular tone and the decline of the eNOS following reperfusion leads to reciprocal vasoconstriction with further deterioration of tissue perfusion. Upregulation of iNOS in response to inflammatory stimuli leads to a second surge of NO three hours following reperfusion.

iNOS becomes upregulated in response to inflammatory stimuli such as endotoxins, cytokines and lipid mediators. The surges in NO production mediated by iNOS are cytotoxic and produced in quantities much greater than other isoforms [18]. iNOS is expressed during inflammation by many types of cells, including hepatocytes.

During I/R injury there is a substantial increase in superoxide production from eNOS as a result of tissue depletion of both L-arginine and the cofactor BH_4_ [19]. As discussed above, superoxide is produced in abundance. Superoxide reacts with the available NO to form peroxynitrite (ONOO^−^), which is pronated to form peroxynitrous acid (HOONO) [20]. Subsequent dissociation of peroxynitrous acid yields highly cytotoxic species NO_2_ and OH·.

The overexpression of eNOS in mice with increased levels of NO by eNOS display protection against I/R injury. Levels of superoxide were reduced by ~50% in skeletal muscle I/R injury [21]. In this model, eNOS-derived NO reduced I/R-induced expression of intercellular adhesion molecule-1 (ICAM-1) and vascular adhesion molecule-1 (VCAM-1) on the endothelium, reducing neutrophil adhesion and margination, thus reducing the severity of tissue damage [21]. 

Proinflammatory cytokines induce the expression of iNOS which produces large quantities of NO. iNOS deficient mice have the capacity to withstand prolonged periods of ischaemia before similar degrees of skeletal muscle necrosis occurs [22]. 

In comparison to NO, endothelin is a potent peptide vasoconstrictor produced by the vascular endothelium. Hypoxia, growth factors, angiotensin II and noradrenaline all stimulate the production of endothelin. Endothelin is increased during I/R injury [23]. As discussed above, the level of NO is greatly reduced in the initial period of reperfusion, with the usual balance between ET/NO shifted towards ET with a subsequent harmful impact on the hepatic microcirculation [24]. The narrowing of liver sinusoids with further hypoxia and local tissue damage occurs. The administration of Bosentan, an endothelin inhibitor, improves tissue viability during reperfusion and highlights its role in I/R injury [24].

Poor hepatic microcirculation has been associated with severe I/R injury in the clinical setting. A study of 566 patients who underwent liver transplant showed that reduced flows in the hepatic artery and portal vein are associated with I/R injury, and can be used to differentiate mild and severe cases [25]. Hepatic blood flow is an important criteria in the viability assessment criteria used during machine perfusion of human livers for transplantation [6].

### 2.3. Cytokines

Hypoxia and I/R injury induce the expression of numerous cytokines, including tumour necrosis factor-α (TNF-α), interleukin-1 (IL-1), interleukin-6 (IL-6), interleukin-8 (IL-8), and platelet activating factor (PAF), in association with elevations in activity of the transcription factor, NF-κB. These cytokines are released systemically and play a role in the development of systemic inflammatory response syndrome (SIRS) and multiorgan dysfunction syndrome (MODS).

TNF-α is a pro-inflammatory cytokine produced by activated macrophages, monocytes, T-lymphocytes, NK cells, and fibroblasts. It is a potent chemoattractant and early response cytokine, which subsequently induces the expression of IL-1, IL-6, IL-8, and PAF. TNF-α is involved in the generation of ROS and enhances the susceptibility of the vascular endothelium to neutrophil mediated injury, by inducing ICAM-1, which mediates the binding of neutrophils to the activated endothelium.

### 2.4. Eicosanoids

The liver is an important organ in the production of eicosanoids [26]. They have a protective role over the liver and attenuate damage caused by complements, endotoxins, and ischaemia amongst other things [27,28,29,30]. Prostaglandins have a protective vasodilatory effect in I/R injury. Due to their short half-life prostaglandins are quickly depleted with a subsequent vasoconstriction with further reduction in blood flow and transport of essential molecules. Exogenous prostaglandin supplementation has been studied in transplantation, showing an improvement in hepatic-splanchnic oxygenation, however, without postoperative graft or biochemical improvement [31]. 

### 2.5. Kupffer Cells and Neutrophils

Kupffer cells (KCs) are hepatic macrophages and reside in the liver sinusoids with sinusoidal endothelial cells, hepatic stellate cells, and dendritic cells. KCs have a significant role in the development of I/R injury. KC activity and its stimulation have been shown to actively worsen I/R injury, whilst its suppression ameliorates injury [32]. KCs generate ROS continuously throughout the reperfusion period by activation of the complement cascade [33,34]. Activated KCs produce proinflammatory cytokines, including IL-1B (interleukin-1-beta) and TNFa [35]. These proinflammatory cytokines stimulate an increased expression of ICAM-1 and VCAM-1 on the surface of hepatocytes and SECs [36,37]. This leads to the activation and migration of CD4+ T-cells and neutrophils [38]. Neutrophils bind to ICAM-1 and VCAM-1 and enter the liver parenchyma. Extravasation into the parenchyma is the prerequisite for neutrophil cytotoxicity [39]. 

The complicated cytokine cascade following I/R injury begins with the upregulation of IL-12 and IL-23. It has been theorised that the origin of these initial cytokines is from KCs and stellate cells [40]. Blockade of these cytokines impacts the expected rise of TNF-α and IFN-gamma, typically seen following I/R injury with less liver injury seen [41,42].

TNF-α is described as the most important mediator in the hepatic inflammatory response to I/R injury [43]. TNF-α acts in a variety of ways including the stimulation of hepatocytes and KCs to produce neutrophil chemoattractants, particularly CXC chemokines [44], upregulates adhesion molecules (ICAM-1, VCAM-1, P-selectin) [45]. The inhibition of TNF-α ameliorates I/R injury [4,45].

### 2.6. Complement

The complement cascade is an important component of inflammation and immune defence. Complements are circulating proteins stimulated by cellular proteins released during reperfusion. Animals with a reduced complement ‘ability’ were found to suffer less severe injury during reperfusion [46]. 

## 3. Machine Perfusion

Machine perfusion as a method of preservation was first utilised by surgeons in renal transplantation [47]. Machine perfusions consist of several approaches and according to the perfusion temperature can be divided into normothermic perfusion (NMP), hypothermic perfusion (HMP), and sub-normothermic perfusion. 

NMP allows the storage, preservation, and transportation of donor organs at physiological temperatures (37 degrees Celsius) under simulated physiological conditions. Normothermic machine perfusion involves the cannulation of arterial inflow (hepatic artery) and venous inflow (portal vein) with continuous blood flow. Blood or acellular oxygen carrier is used as the perfusion fluid, with an oxygenator in situ and pressure monitors. The rationale behind this technique being that under these physiologic conditions the organ’s metabolic functions can continue and the damage caused by cold and warm ischaemia can be limited and, possibly, reversed. Additional benefits include the possibility of functional testing of the organ prior to transplantation and improved assessment by the transplantation team [6]. Mergental et al. have shown that initially discarded livers can be optimised using NMP, with strict viability testing, with successful transplantation of 71% previously discarded livers with 100% 90-day patient and graft survival [6]. Preservation of the organ is, ideally, the shortest possible time, taking into account transportation, logistics and explantation (and viability testing as required), but the preservation of a liver under normothermic conditions has been prolonged as long as 7 days [48]. NMP requires an oxygen carrier as perfusion fluid and successful perfusions have been achieved, utilising both blood and nonblood based acellular oxygen carriers [49]. The use of NMP was first trialled clinically in 2016 and showcased the clinical feasibility of the technique with 100% of grafts surviving 6 months [50]. 

Hypothermic machine perfusion (HMP) is an alternative method of organ preservation utilised for the past 60 years, initially focusing on renal transplantation but has proved to be effective for the transplant of other solid organs [51]. Where NMP aims to replicate the physiologic conditions with blood-based perfusate and normothermic temperatures, HMP utilises artificial perfusion solutions with or without oxygenation under hypothermic conditions (4–11 degrees Celsius). Multiple clinical trials in humans have shown the success of orthotopic liver transplantation following both NMP and HMP/HOPE treatment.

Studies comparing machine perfusion with the SCS of liver grafts are limited in comparison to kidney transplantation due to its recent popularity. Rates of primary nonfunction (PNF) and delayed graft function (DGF) in kidney grafts undergoing HMP have been shown to be lower compared to those preserved with SCS [52,53,54,55]. These results have been replicated using HMP in liver grafts; a systematic review of 7963 patients displayed a lower prevalence of DGF in organs preserved with HMP compared to SCS [56]. The use of HMP in liver preservation has shown improvement in early allograft dysfunction and postoperative parameters, such as bilirubin and AST levels, compared to SCS [57,58]. The rate of postoperative biliary strictures has also been shown to be lower in HMP preserved organs [57,58,59,60]. Studies with NMP are limited but have shown lower incidence of early allograft dysfunction compared to SCS [50,61,62]. 

Machine perfusion circuits contain oxygenators to oxygenate blood through the hepatic artery and portal veins. Oxygen carriers are required in the form of either packed red cells or an acellular oxygen carrier [49]. While they provide a suitable carrier for transport and delivery of oxygen, they lack some key components found in human blood such as platelets, plasma, and immune cells. I/R injury leads to the activation of both the innate immune system and the adaptive immune system. It can be prophesised that machine perfusion may limit the extent of I/R in the absence of the body’s systemic immune system. It has even been shown that packed red cells may have an immunosuppressive effect, with monocytes incubated in stored packed red cells producing less TNFa on stimulation [63]. There is no data examining the possible benefits of machine perfusion in exposing the organ to ischaemia reperfusion in the absence of systemic immune cells but it may be a possible avenue of future research.

### 3.1. Gene Expression

Alteration in gene expression has been shown to be augmented during NMP. Jassem et al. compared allografts using NMP and SCS in 2013 [64]. Despite postoperative parameters being comparable between the two cohorts, including biomarkers and patient outcomes, however, there was significant difference in gene expression [64]. Alteration in gene expression was compared between the two groups, with the number of genes downregulated outnumbering those upregulated in both groups. Of those upregulated in the NMP group, the genes were related to tissue regeneration and platelet function, but a few related to immune cell function. Within the SCS cohort, however, there was a high representation of proinflammatory cytokines and genes involved in humoral immunity, neutrophil chemotaxis and platelet function were upregulated. In comparison, NMP showed downregulation of pathways, including allograft rejection, graft versus host disease, immune pathways, programmed cell death protein 1 (PD1) signalling, IL-2, IL-12, IL-6, C-C chemokine receptor type 5 preperfusion (post-NMP, pretransplant). 

### 3.2. Autophagy

Autophagy is a cellular pathway crucial in the survival and homeostasis of cells [65,66]. Autophagy eliminates damaged organelles, long-lived proteins or intracellular pathogens through the coordinated engulfment of the targeted cargo in a double membrane cytoplasmic structure known as an autophagosome [67,68]. There are three main types of autophagy, of which macro-autophagy is the most common. Autophagy has a prosurvival role in hepatocytes undergoing I/R injury, providing a coping mechanism. ROS are an important regulator of autophagy activity, with the accumulation of intracellular ROS associated with autophagy in hepatocytes. Inhibition in the production of ROS leads to the inhibition of autophagy in human hepatocytes and increased rates of apoptosis [69].

Calcium signalling has been theorised to regulate autophagy activation in NMP. During NMP, the perfusate is supplemented with calcium to maintain physiological extracellular levels of the ion ensuring that the electrochemical gradient of calcium is maintained across cell membranes [70]. Low intracellular levels of calcium are reported to induce CaMMk-Beta activation following mTOR inhibition and therefore ULK1/autophagy activation [71]. Physiological calcium levels during NMP potentially promote normal autophagy activity within liver sinusoidal endothelial cells and other liver cells ensuring maintenance of homeostasis. The mechanical manipulation of autophagy during NMP is dependent on shear stress. NMP has the advantage of providing an adjustable vascular/laminar flow rate to livers. In turn this flow rate provides a near physiological shear stress known to promote autophagy induction [72]. Activated autophagy in endothelial cells is fundamental for eNOS transcription, NO production and maintenance of vascular tone maintenance [73]. The increased presence of NO reduces platelet aggregation and leukocyte adhesion in endothelial cells thus reducing IRI and hepatic microcirculatory disturbance [74]. Pharmacological modulation of autophagy during NMP offers the opportunity to target endothelial cells and their dysfunction prior to transplantation of the organ. Extended criteria liver donors are associated with lower cellular ATP content and increased ROS production increasing their susceptibility to I/R injury [75,76]. Pharmacological agents promoting the activation of autophagy during NMP may promote the elimination of damaged organelles and toxins prior to graft implantation. Aiding mitophagy function may be crucial to maintaining liver sinusoidal endothelial cell function during NMP and thus aiding the survival of other liver cells. As described above, many drugs are already known to regulate autophagy activation and the use of these drugs in experimental NMP perfusion offers an important method to evaluate these interventions.

### 3.3. Hypothermic Oxygenated Machine Perfusion

Hypothermic Oxygenated Machine Perfusion (HOPE) is an evolution of HMP with active oxygenation of the perfusion fluid throughout the procedure, aiming to provide 60−100 kPa of oxygen to the perfused organ [77]. 

Additional oxygenation of the perfusion fluid and its benefits were first identified in renal transplantation utilising HMP, and the results were found to be true for liver perfusion [78,79,80]. Oxygen is an essential component of successful perfusion. Grafts perfused with nitrogenated perfusion fluid (pO2 < 2 kPa) led to the death of all grafts within 28 days, with evidence of severe I/R injury and acute graft failure [79]. The protective effects exhibited by HMP are oxygen-dependent and are critical in reducing the production of ROS and protecting mitochondria. 

There is evidence to support HOPE reducing immunosuppressant requirement post-transplantation. At 4 weeks post-transplantation, patients that received a graft preserved by HOPE (without immunosuppression) exhibited evidence of graft injury comparable to that of the sub-therapeutic immunosuppression group (utilising SCS), however evidence of hepatocellular injury, I/R injury, and T cell infiltration of the graft was reduced [79]. The team later expanded on this, comparing HOPE grafts with non-HOPE grafts, both immunosuppressed with low-dose tacrolimus. All recipients of the HOPE arm survived 28 days without evidence of rejection [79]. The authors theorised that HOPE treatment may lead to lower immunosuppression requirements postoperatively without an increased threat of rejection [79]. 

Autophagy has been shown to be a protective benefit of HOPE [81]. Utilising a rat model, Zeng et al. compared HOPE and hypothermic deoxygenated nitrogenated perfusion (HNPE) [81]. Organs perfused with HOPE had evidence of higher tissue ATP content and higher expression of immune factors associated with activated autophagy (LC3B-II, autophagy-related 5, uncoordinated 51-like kinase 1) [81]. These markers were independent to that of the HOPE cohort, indicative of HOPE enhancing autophagy. This protective effect was lost on administration of 3-methyladenine (3-MA), an autophagy inhibitor, with no upregulation of markers indicative of autophagy. This 3-MA group displayed worse liver function, higher oxidative stress levels and more necrotic cells comparative to HOPE without 3-MA. Autophagy is therefore a protective benefit of HOPE.

### 3.4. Mitochondrial Respiratory Complex I

Mitochondrial respiratory Complex I (also known as ubiquinone oxidase) is the first enzyme in the mitochondrial electron transport chain and catalyses the transfer of electrons from NADH to Ubiquinone (Co-enzyme Q). Complex I has been described as the rate-limiting step in overall respiration and is considered an essential component of energy metabolism [82]. Further to its role in oxidative phosphorylation, it has an important role in the regulation of ROS [82]. Under normal conditions, Complex I catalyses the oxidation of NADH, reducing flavin mononucleotide (FMN) to form FMH2. This reaction transfers electrons to Ubiquinone [83]. Respiratory Complex II (succinate ubiquinone oxidoreductase) catalyses the oxidation of succinate to fumarate, and in doing so, transfers electrons to the quinone pool.

During reperfusion, superoxide is produced. Complex I is able to produce ROS under two conditions: during the normal forward electron transport during the reduction of NADH; and during reverse electron transport (RET), where electrons are forced backwards through Complex I when succinate is used as Complex II substrate. Inhibition of Complex I has been shown to reduce ROS produced during reperfusion, and so, forward electron transport is unlikely to be a major component of I/R injury [84]. However, the reduction in ROS seen during Complex 1 inhibition may also be due to a reduction in RET-induced ROS generation and a reduction in ROS produced by Complex III. RET, however, can drive superoxide formation—electrons flow backwards through Complex I and onto FMN where they reduce NAD^+^ to NADH [84].

A study of I/R injury in brain tissue has theorised that the mitochondrial production of ROS may be accelerated if there is an accumulation of succinate, and when reperfusion restarts the mitochondrial oxidises succinate preferentially [85]. Further studies exploring this have shown that oxidation of succinate under RET conditions results in a release of flavin from Complex I [86]. This further alters the oxidation of NAD-linked substrates further compounding the release of ROS [86]. 

Succinate accumulation and FMN release from mitochondria during reperfusion are key events in the release of mitochondrial ROS. Schlegel at al. explored mitochondrial injury following I/R injury, comparing HMP and NMP. Following a period of warm ischaemia, rodent livers were exposed to various doses of succinate, and measuring mitochondrial and metabolic parameters. 

Schlegel et al. using HOPE and NMP, observed that FMN release was three- to eight-fold lower in the HMP group [80]. This effect was observed with and without succinate exposure. The team showed that at normothermic temperatures uptake of succinate from the perfusate was not different. They observed a dose-dependent rise in FMN as perfusate FMN increased. Perfusate FMN was correlated with perfused NAD/NADH and IMP. On analysis of mitochondrial FMN, the results were the inverse to those seen in the perfusate, suggesting mitochondrial origin. The group went on to compare FMN release from mitochondria in both NMP and HMP, and they found that at hypothermic temperatures (10 degrees Celsius), there was evidence of lower levels of inflammation, even in the presence of excess succinate in the perfusate. Following this, the Zurich group found evidence that subjecting rat DCD livers to HOPE followed by NMP improved the function of mitochondrial respiratory complexes and effectively induced mitochondrial reprogramming [80].

### 3.5. Nrf2-Antioxidant Response Element Signalling Pathway

The Nrf2-antioxidant response element signalling pathway is a mechanism of defence against oxidative stress at the cellular level and acts to detoxify and eliminate ROS by enhancing gene expression and increasing antioxidant capacity [87]. Xue et al. theorised that HMP may reduce I/R injury by activation of the Nrf2-antioxidant response element signalling pathway [88]. The study measured markers of oxidative stress during HMP and SCS groups in addition to the expression of Nrf2. In the HMP group, markers of oxidative stress were reduced, associated with the increased expression of Nrf2. The reverse was seen in the SCS group. Following reperfusion, antioxidants associated with the Nrf2 pathway (GST-1, NQO1, and GCL) were increased in the HMP group.

### 3.6. Transcription Factor NF-κB

Transcription factor NF-κB increases the expression of proinflammatory cytokines, acute phase proteins, and cell adhesion molecules and is an important mediator of I/R injury. 

There are two main pathways for NF-κB activation following I/R injury. In unstimulated cells, NF-κB is sequestered in the cytoplasm by binding to IkB proteins. IkB proteins mask the nuclear localisation sequence of NF-κB, thereby preventing its translocation into the nucleus. In classical pathways, cell stimulation results in the serine phosphorylation of IkB by the IkB kinase complex (IKK complex). Phosphorylated IkB then becomes the target of ubiquitin ligase, which polyubiquitinates the protein for subsequent proteasomal degradation [89,90]. Another pathway for the activation of NF-κB involves the phosphorylation of IkB-α on tyrosine residue 42 that leads to its dissociation from NF-κB (58). In both pathways, once NF-κB is free from IkB, it translocates to the nucleus where it binds DNA and initiates the transcription of target genes. 

During the initial phase of injury, NF-κB is activated by oxidant stress and proinflammatory stimuli to increase the expression of proinflammatory cytokines, chemokines, and adhesion molecules. In Kupffer cells, NF-κB activation promotes the expression of TNF-α and IL-6 after I/R injury [91]. In hepatocytes, NF-κB activation drives their production of TNF-α [92]. NF-κB activation in endothelial cells leads to the expression of chemokines of the IL-8 family and the adhesion molecules E-selectin, ICAM-1, and VCAM-1 [93,94]. 

Ramachandran et al. theorised that inhibition of this pathway would reduce the effects of I/R injury in steatotic liver tissue [95]. Compared to lean liver tissue, fatty livers demonstrated increased levels of NF-κB postperfusion (46). Administration of an FDA approved inhibitor of NFkB, Bortezomib, reduced the level of expression seen in fatty liver grafts [95]. 

HMP has been observed to cause a significant reduction in the expression of NFkB compared to traditional SCS [96]. Sirtuin-1 (SIRT-1) is a deacetylase that has been shown to inhibit NF-κB and its use is associated with a reduction in I/R injury. Levels of SIRT-1 in HMP grafts were significantly raised in HMP grafts compared to SCS, consistent with SIRT-1 mediated deacetylation of NF-κB [96]. A reduction of downstream proinflammatory cytokines (IL-6 and TNFa) was seen, and the team theorised that hypothermic machine perfusion attenuates the inflammatory response induced by hepatic NFkB (in a rat model) [96]. 

## 4. Conclusions

Most of the research into machine perfusion over the past decade has been establishing the efficacy and clinical safety of liver perfusion and translating this into clinical practice. 

The advancements in machine perfusion for liver transplantation have allowed for the successful utilisation of extended criteria donors providing a future avenue to combat the increasing length of transplant waiting lists. Despite its significant successes, machine perfusion remains in its relative infancy. Elucidating the individual mechanisms behind each type of machine perfusion is a crucial next step in advancing machine perfusion technology and we are confident that great strides will take place in the near future. Analysing ischaemia reperfusion injury and how machine perfusion augments its process allows further research into how best to optimise, augment and reduce the damage caused by ischaemia reperfusion injury and further increase the number of available organs for transplantation. 

This review set out to identify the mechanisms behind how the various types of machine perfusion ameliorate ischaemia reperfusion injury. There is very sparse information on the mechanism behind the benefits seen in normothermic machine perfusion, comparative to hypothermic machine perfusion. The assumed mechanism behind its benefits is its minimisation of anaerobic metabolism during cold ischaemia with a reduction in the accumulation of toxic byproducts and the restoration of ATP. The precise mechanisms, however, remain unknown. 

The use of ischaemia-free organ transplantation has been hypothesised and confronts the seemingly unavoidable graft dysfunction caused by ischaemia reperfusion injury. A successful ischaemia-free liver transplant was performed by He et al. in 2017 [97]. Proinflammatory cytokines showed no change compared to pre-perfusion, no significant hepatocyte necrosis or apoptosis was noted [97]. This study has highlighted a possible benefit of normothermic machine perfusion of almost avoiding the reperfusion process by eliminating warm ischaemia (and cold ischaemia). 

Ischaemia reperfusion injury is a complex process, with the involvement of many cell types and pathways. The number of patients on the liver transplant list increases year on year and the number of liver grafts available for donation unfortunately does not match this need. Machine perfusion has opened an avenue to optimise suboptimal grafts and extend the available grafts with the aim of reducing waiting lists and reducing the morbidity and mortality of those on the list.
